# Race and Urbanity Alter the Protective Effect of Education but not Income on Mortality

**DOI:** 10.3389/fpubh.2016.00100

**Published:** 2016-05-20

**Authors:** Shervin Assari, Maryam Moghani Lankarani

**Affiliations:** ^1^Department of Psychiatry, University of Michigan, Ann Arbor, MI, USA; ^2^Center for Research on Ethnicity, Culture and Health, School of Public Health, University of Michigan, Ann Arbor, MI, USA; ^3^Medicine and Health Promotion Institute, Tehran, Iran

**Keywords:** ethnic groups, Blacks, Whites, socioeconomic status, education, income, urbanity, mortality

## Abstract

**Background:**

Although the effects of socioeconomic status (SES) on mortality are well established, these effects may vary based on contextual factors such as race and place. Using 25-year follow-up data of a nationally representative sample of adults in the U.S., this study had two aims: (1) to explore separate, additive, and multiplicative effects of race and place (urbanity) on mortality and (2) to test the effects of education and income on all-cause mortality based on race and place.

**Methods:**

The Americans’ Changing Lives (ACL) Study followed Whites and Blacks 25 years and older from 1986 until 2011. The focal predictors were baseline SES (education and income) collected in 1986. The main outcome was time until death due to all causes from 1986 until 2011. Age, gender, behaviors (smoking and exercise), and health (chronic medical conditions, self-rated health, and depressive symptoms) at baseline were potential confounders. A series of survey Cox proportional hazard models were used to test protective effects of education and income on mortality based on race and urbanity.

**Results:**

Race and place had separate but not additive or multiplicative effects on mortality. Higher education and income were protective against all-cause mortality in the pooled sample. Race and urbanity significantly interacted with baseline education but not income on all-cause mortality, suggesting that the protective effect of education but not income depend on race and place. While the protective effect of education were fully explained by baseline health status, the effect of income remained significant beyond health.

**Conclusion:**

In the U.S., the health return associated with education depends on race and place. This finding suggests that populations differently benefit from SES resources, particularly education. Differential effect of education on employment and health care may explain the different protective effect of education based on race and place. Findings support the “diminishing returns” hypothesis for Blacks.

## Introduction

The protective effects of socioeconomic status (SES; e.g., education and income) on health are consistently shown (1). Mirowsky and Ross described the effect of SES on health as “enduring, consistent, and growing” (2). State-of-the-art longitudinal studies, such as the Health and Retirement Study (HRS) (3), Americans’ Changing Lives (ACL) Study (4), Panel Study of Income Dynamics (5), British Cohort Study (BCS) (6), the British Whitehall Study (7), French GAZEL cohort (7), and Survey of Health, Aging and Retirement in Europe (SHARE) (8), have all shown that higher levels of education and income are associated with lower levels of morbidity (4) and mortality (9). Higher education and income enable individuals to avoid risks and minimize negative consequences as they occur (10, 11).

In the U.S., the education system and job market are both segregated by race, place, and social class (12, 13). As a result, even if they not drop out of school, Blacks do not have the same chance of receiving high-quality education (14, 15) or high-paying jobs (16, 17) as Whites. Thus, instead of having similar health effects across race, place, and social class (18), education and income may have differential protective effects across sub-populations (19, 20). If the school system is less resourced where Blacks live, the same education degree may have smaller effects on human capital for Blacks compared to Whites (14, 15). In addition, race also alters the effect of educational credentials on health through labor market advantages for Whites (21). However, it is still unknown if health gains associated with education and income depend on race and place (22–24).

Our knowledge of the effect of place on health is mostly limited only to the direct effect (25, 26). Research has shown that place influences health beyond individual-level factors (27) by conferring additional risk beyond individual SES status (28). Sastry argues that although SES partly explains why place (rural versus urban) causes disparities in health, interactions between SES and place also exist (29). As the neighborhood effects literature has mostly focused on the main effect of place on health (30), a question yet to be answered is whether place also alters the effects of SES resources on health. We still do not know whether protective effects of SES on health depend on place (31, 32). This research conceptualizes place as a moderator (33), which requires testing statistical interaction between place and social class (28). Very few studies have tested if the effects of race and place on disparities are independent, additive, sub-additive, or synergistic (34, 35).

This study investigated separate, additive, and multiplicative effects of race, place, and SES (education and income) on mortality. We were particularly interested in the moderating effects of race and place on the protective effects of baseline education and income (in 1986) on all-cause mortality during a 25-year follow-up in the U.S. We had three specific hypotheses: first, race and place would have separate, additive, and multiplicative effects on mortality. Second, the effects of education and income on mortality would be weaker for Blacks compared to Whites. Third, we expect urbanity to modify the effect of education and income on all-cause mortality. To provide generalizable results to the U.S., we used a nationally representative sample of American adults.

## Materials and Methods

The ACL is a 25-year cohort study conducted from 1986 to 2011. The ACL study is the oldest ongoing longitudinal study with a nationally representative sample. The aim of the study is to investigate the role of a broad range of social, environmental, psychological, and behavioral factors (along with medical care) in health changes with age over the life course. The study began in 1986 with a national face-to-face survey of 3,617 adults ages 25 and up in the continental U.S. (36–41).

### Participants

The ACL has enrolled a nationally representative sample of non-institutionalized U.S. adults (*n* = 3,617) aged 25 and older in 1986 (household response rate = 70%, individual response rate = 68%). Both individuals over 60 years of age and Blacks were oversampled (36–41). (Analytical sample = 3,361, composed of 2,205 Whites and 1,156 Blacks).

### Process

Although four follow-up interviews were conducted with respondents, our analysis is restricted to baseline data and survival follow-up, neither of which depended on successful follow-up of respondents to the survey.

### Measures

Demographic and SES characteristics were obtained from wave 1 interviews. Demographic covariates included gender (0 = male, 1 = female), race (0 = White, 1 = Black), and age in years (a continuous variable).

### Race

Participant’s race was defined based on self-reported race and ethnicity, collected at baseline in 1986 with several survey items. Respondents gave an open-ended response to the question, “In addition to being American, what do you think of as your ethnic background or origins?” Respondents were then asked a multiple-choice question, “Are you white, black, American Indian, Asian, or another race?” and allowed to answer with multiple categories. Those who responded with more than one non-white group were asked to identify which “best described” their race. The survey also assessed the state or foreign country in which the respondent, respondent’s mother, and respondent’s father were born, and the respondent’s father’s last name. Finally, participants were asked “Are you of Spanish or Hispanic descent, that is, Mexican, Mexican–American, Chicano, Puerto Rican, Cuban, or Other Spanish?” Responses from the above questions were used to construct race categories of “Non-Hispanic White,” “Non-Hispanic Black,” “Non-Hispanic Native American,” “Non-Hispanic Asian,” and “Hispanic.” This study’s analysis only included Non-Hispanic White and Non-Hispanic Black respondents (38–41).

### Urbanity

We collected information on urbanity at an individual level. We defined urbanity as a three-level categorical variable. Multiple items were used to define urbanity in this study. Categories included (1) inner cities (urban areas), defined as the centers (large or small) of cities, which are classified as metropolitan statistical areas greater than or equal to 50,000 people; (2) suburban areas, defined as suburbs to large or small central cities, and (3) rural areas, defined as other adjacent and outlying areas. Our urbanity measure is comparable with the Rural-Urban Continuum Codes, developed by the USDA Economic Research Service (42). This system distinguishes metropolitan (metro) counties by the population size of their metro area, and non-metropolitan (non-metro) counties by degree of urbanization and adjacency to a metro area or areas (42). These categories have major implications for capturing access to health-care services (43).

### Education

Education, measured in 1986 as years of completed education, was the first SES predictor of interest. Due to low frequency of tertiary education among Blacks in 1986, we operationalized education in two ways: a continuous measure, reflecting years of education, and a three-level categorical variable (<12 years, 12 years, and >12 years). Other studies have operationalized education similarly (44–47).

### Income

We measured baseline income (respondent and spouse total income) in 1986 as our second SES predictor of interest.

### Chronic Medical Conditions

Number of chronic medical conditions (CMC) was measured using self-report data at baseline (1986). All participants were asked whether a health-care provider had ever told them they had any of the following seven focal conditions: hypertension, diabetes, chronic lung disease, heart disease, stroke, cancer, and arthritis. A sum score was calculated, ranging from 0 to 7. A detailed description on the measurement of CMC is provided elsewhere in House and colleagues (37).

### Self-Rated Health

Respondents were asked to rate their self-rated health (SRH) as excellent, very good, good, fair, or poor. The literature has treated the SRH in three distinct ways, namely as a dichotomous variable, a nominal variable, and a continuous score (48–51). We treated SRH as a dichotomous variable. We collapsed it into two categories (fair/poor vs. excellent/very good/good), a cutoff point that is common in the literature (38).

### Depressive Symptoms

Depressive symptoms were measured with an 11-item version of the Center for Epidemiological Studies-Depression scale (CES-D) (52). CES-D items measure the extent to which respondents felt depressed, happy, lonely, sad, that everything was an effort, that their sleep was restless, that people were unfriendly, that they did not feel like eating, that people dislike them, that they could not get going, and that they enjoyed life. Positively worded items were reverse coded. This abbreviated CES-D scale has shown acceptable reliability and a similar factor structure compared to the original version (53–55). Possible item responses were scored 1 (never or hardly ever) to 3 (most of the time). We used an average score of depressive symptoms, conceptualized as a continuous measure with a potential range from 1 to 3. Higher scores indicated greater severity of depressive symptoms (39–41).

### Exercise

A physical activity index was derived from answers to survey questions regarding engagement in exercise, active sports, gardening/yard work, household chores, and walking. Higher scores on this index were indicative of more exercise frequency (36, 56).

### Smoking

Information was collected on self-reported history of smoking. We used a dichotomous variable (current smoker = 1, never or ex-smoker = 0).

### All-Cause Mortality

The main outcome variables were mortality from all causes, internal causes, and external causes. Information on all deaths from mid-1986 through 2011 was obtained through the National Death Index (NDI), death certificates, and also from informants. In most cases, time and cause of death were verified with death certificates. The handful of cases where death could not be verified with death certificates were reviewed carefully, and actual death was certain in all cases. Only in these cases, lacking death certificates was the date of death ascertained from the informants or the NDI report (39–41, 57, 58).

### Statistical Analysis

Due to the complex sample design used in the ACL, Stata 13.0 (Stata Corp., College Station, TX, USA) was used for data analysis. Application of baseline weights provides rates and results that are generalizable to the US population in 1986. Taylor series linearization was used for estimation of SEs.

We estimated seven Cox proportional hazard models to determine the effects of education and income on mortality. Baseline SES (education and income) collected in 1986 was the focal predictor, time to death through 2011 from all causes was the main outcome, and age, gender, and health (CMC, SRH, and depressive symptoms) were confounders. Hazard ratios (HRs) with 95% confidence intervals are reported. A HR of <1 indicates a protective effect of the independent variable on the outcome. Because we consider multiple outcomes, we set *p* < 0.01 as the level of statistical significance. For comparability, all main effects and interaction terms were left in models if they were not significant.

*Model 1* estimated age- and gender-adjusted effect of race. *Model 2* tested age- and gender-adjusted effects of place. *Model 3* estimated additive (combined) effects of race and place. *Model 4* also controlled for SES, to test if education and income explain the effect of race and place on mortality. *Model 5* also controls for health, in addition to SES. *Models 3–5* also included interactions between race and place to test multiplicative effects of race and place. *Model 6* and *Model 7* included interaction terms between race and place with education and income. While *Model 6* did not include health status, *Model 7* controlled for baseline health.

## Results

Table 1 shows descriptive statistics for the analytic sample overall and by race. While there were no race differences in age or gender (*p* > 0.05 for both comparisons), average education (12.69 years for Whites and 11.37 years for Blacks, *p* < 0.001) and income (USD 5,570 for Whites and USD 4,250 for Blacks, *p* < 0.001) were lower for Blacks than Whites. Mortality was higher among Blacks (39.41%) compared to Whites (35.66%) (*p* < 0.05).

**Table 1:** **Distribution of the sample characteristics by race**.

	All	White	Black	
	Mean (95% CI)	Mean (95% CI)	Mean (95% CI)	*p*
Age	47.77 (46.69–48.84)	47.96 (46.75–49.17)	46.33 (44.89–47.78)	0.093
Education (years)	12.53 (12.34–12.73)	12.69 (12.48–12.90)	11.37 (10.90–11.84)	<0.001
Income (USD 1,000)	5.41 (5.22–5.60)	5.57 (5.36–5.77)	4.25 (3.88–4.62)	<0.001
Gender (female)	51.66 (48.75–54.56)	50.96 (47.67–54.24)	56.38 (51.61–61.04)	0.086
Education (11 years or less)	23.96 (21.40–26.74)	21.73 (18.88–24.88)	40.48 (34.79–46.44)	<0.001
Death (all cause)	36.11 (33.52–38.78)	35.66 (32.77–38.67)	39.41 (35.29–43.68)	<0.05

Table 2 reports results of five Cox proportional hazard models in the pooled sample. *Model 1* reports the age- and gender-adjusted effect of race. Based on this model, race had a significant age- and gender-adjusted effect on mortality (HR = 1.42, 95% CI = 1.25–1.62). *Model 2* reports age- and gender-adjusted effects of place. Based on Model 2, place also had a separate effect on mortality risk. Based on this model, compared to residing in the inner city, living in suburban areas (HR = 0.85, 95% CI = 0.70–1.03) was marginally associated with lower mortality risk, while residence in rural areas (HR = 0.80, 95% CI = 0.65–0.98) was significantly associated with lower mortality risk. *Model 3* reports the additive effects of race and place. Based on this model, the effect of place on mortality did not remain above and beyond race (*p* > 0.05 for both living in suburban and rural areas). *Model 4* also controls for SES and shows that education and income explain the effect of race on mortality. In this model, education (HR = 0.80, 95% CI = 0.71–0.91) and income (HR = 0.91, 95% CI = 0.89–0.94) were protective against mortality. *Model 5* also controls for health, as well as SES. Based on this model, the effect of education on mortality is explained by health (*p* > 0.05). However, the effect of income on mortality stays in the model (HR = 0.94, 95% CI = 0.91–0.97). Based on *Models 3–5*, race and place did not have multiplicative effects, as interaction terms between race and urbanity were non-significant (*p* > 0.05 for all interactions terms).

**Table 2:** **Results of Cox proportional hazard models on the separate and additive and multiplicative effects of race and place on mortality**.

	HR (SE)	95% CI	HR (SE)	95% CI	HR (SE)	95% CI	HR (SE)	95% CI	HR (SE)	95% CI
	
	Model 1		Model 2		Model 3		Model 4		Model 5	
Race										
Whites	Ref		–	–	ref		ref		ref	
Blacks	1.42 (0.09)***	1.25–1.62	–	–	1.26 (0.12)*	1.03–1.53	1.08 (0.10)	0.90–1.29	0.98 (0.09)	0.81–1.18
Place										
Inner city	–	–	ref		ref		ref		ref	
Suburban	–	–	0.85 (0.04)^#^	0.70–1.03	0.99 (0.15)	0.72–1.34	0.99 (0.15)	0.73–1.35	1.09 (0.15)	0.82–1.44
Rural	–	–	0.80 (0.08)*	0.65–0.98	1.01 (0.13)	0.78–1.31	0.87 (0.11)	0.67–1.12	0.88 (0.13)	0.65–1.20
Gender										
Male	ref		ref		ref		ref		ref	
Female	0.61 (0.04)***	0.53–0.69	0.60 (0.04)***	0.53–0.69	0.60 (0.04)***	0.53–0.68	0.55 (0.04)***	0.48–00.63	0.52 (0.04)***	0.45–0.60
Age	1.09 (0.00)***	1.09–1.10	1.09 (0.00)***	1.09–1.10	1.09 (0.00)***	1.09–1.10	1.09 (0.00)***	1.08–1.09	1.09 (0.00)***	1.08–1.09
Education										
11 years or less	–	–	–	–	–	–	ref		ref	
12 years or more	–	–	–	–	–	–	0.80 (0.05)**	0.71–0.91	0.91 (0.06)	0.80–1.04
Income	–	–	–	–	–	–	0.91 (0.01)***	0.89–0.94	0.94 (0.01)***	0.91–0.97
Smoking										
Non-smoker	–	–	–	–	–	–	–	–	ref	
Current smoker	–	–	–	–	–	–	–	–	1.78 (0.14)***	1.53–2.07
Exercise	–	–	–	–	–	–	–	–	0.89 (0.03)***	0.84–0.95
Chronic medical conditions	–	–	–	–	–	–	–	–	1.13 (0.03)***	1.07–1.20
Depressive symptoms	–	–	–	–	–	–	–	–	1.00 (0.04)	0.92–1.08
Self-rated health	–	–	–	–	–	–	–	–	1.21 (0.05)***	1.11–1.32
Blacks × Suburban	–	–	–	–	0.90 (0.16)	0.63–1.28	0.92 (0.16)	0.65–1.30	0.86 (0.14)	0.62–1.20
Blacks × Rural	–	–	–	–	0.81 (0.12)	0.59–1.09	0.90 (0.13)	0.67–1.21	0.92 (0.15)	0.66–1.29

*^#^*p* < 0.1*.

*p < 0.05.

**p < 0.01.

***p < 0.001.

Table 3 also shows two models (*Model 6* and *Model 7*) that included interaction terms between race, place, education, and income. While Model 6 did not include health status, *Model 7* also controlled for baseline health. Based on *Model 6*, race moderated the effect of education but not income on mortality. In this model, we found a significant interaction term between race and education (HR = 0.71, 95% CI = 0.50–0.99). This model also showed a marginally significant interaction between place and education (HR = 0.71, 95% CI = 0.48–1.04). In *Model 7*, which also controlled for health, the interaction term between race and education stayed significant (HR = 0.70, 95% CI = 0.50–0.98), but the interaction between place and education was explained by baseline health status (*p* > 0.05).

**Table 3:** **Results of Cox proportional hazard models on the effects of baseline education and income on mortality based on race and place**.

	HR (SE)	95% CI	HR (SE)	95% CI

	Model 6		Model 7	
Race				
Whites	ref		ref	
Blacks	1.50 (0.26)*	1.05–2.14	1.41 (0.22)*	1.03–1.93
Place				
Inner city	ref		ref	
Suburban	1.43 (0.31)	0.92–2.20	1.42 (0.28)^#^	0.96–2.11
Rural	1.07 (0.23)	0.69-–1.65	1.19 (0.27)	0.75–1.90
Gender				
Male	Ref		ref	
Female	0.55 (0.04)***	0.48–0.63	0.52 (0.04)***	0.45–0.61
Age	1.09 (0.00)***	1.08–1.09	1.09 (0.00)***	1.08–1.09
Education				
11 years or less	Ref		ref	
12 years or more	0.62 (0.08)**	0.47–0.82	0.71 (0.10)*	0.53–0.96
Income	0.96 (0.03)	0.89–1.03	0.99 (0.03)	0.93–1.06
Smoking				
Non-smoker	–	–	ref	
Current smoker	–	–	1.77 (0.14)***	1.52–2.07
Exercise	–	–	0.89 (0.03)***	0.84–0.95
Chronic medical conditions	–	–	1.13 (0.03)***	1.07–1.20
Depressive symptoms	–	–	1.00 (0.04)	0.93–1.08
Self-rated health	–	–	1.21 (0.05)***	1.11–1.32
Blacks × Education	0.71 (0.12)*	0.50–0.99	0.70 (0.12)*	0.50–0.98
Suburban × Education	0.71 (0.14)^#^	0.48–1.04	0.73 (0.14)	0.50–1.07
Rural × Education	0.84 (0.16)	0.57–1.23	0.82 (0.15)	0.56–1.18
Blacks × Income	0.98 (0.03)	0.92–1.04	0.97 (0.03)	0.92–1.02
Suburban × Income	0.94 (0.04)	0.86–1.02	0.94 (0.04)	0.87–1.02
Rural × Income	0.95 (0.04)	0.87–1.04	0.94 (0.04)	0.85–1.02

*^#^*p* < 0.1*.

*p < 0.05.

**p < 0.01.

***p < 0.001.

## Discussion

Our study suggests that, in the U.S., the protective effects of education but not income on all-cause mortality is moderated by race and place. Our findings support the *diminishing returns* hypothesis for Blacks. Based on this hypothesis, as education levels increase, Blacks do not gain as much improvement in health as Whites (59–61). This hypothesis also suggests that disparity between Blacks and Whites is largest at the highest but not lowest SES levels (59). While the effect of race on health varies among low versus high SES levels, health effect of SES also depends on race (59, 62).

Previous research has suggested that protective effects of SES indicators against mortality are not necessarily uniform across sub-populations (1, 19, 20, 22, 63). This is possibly because education does not similarly improve life circumstances and does not similarly reduce exposure to risk and protective factors across various social groups (64). We argue that differential access to job and health care as well as differential pay based on race and place may explain differential survival benefits associated with education based on group membership.

Multiple previous studies have shown Black–White differences in the effect of SES indicators on health. A previous study showed a larger protective effect of education against mortality due to all causes as well as internal causes for Whites compared to Blacks^1^. In the National Longitudinal Mortality Study from 1979 to 1998, among non-Hispanic Blacks, there were step reductions in mortality at 12 and 16 years of education, with constant slopes between the steps. For Whites, each additional year of education had a stronger effect on decreasing mortality, but only among those with at least a high school diploma. Whites also showed a step reduction in mortality at 12 years of education (65). In another study on mortality data in the 1960s, education was linked to a lower risk of mortality for Whites but not Blacks (66). Everett and colleagues used the U.S. National Health Interview Survey-Linked Mortality sample and showed that race, gender, and cohort modified the effect of education on mortality, documenting significant changes across birth cohorts in the associations between education and mortality for White women and White men, but no such changes across cohorts of Black men (67). In a study by Hayward, Hummer, and Sasson, Whites showed an effect of education on mortality, but there was almost no association for Blacks in that period (66). Backlund and colleagues found that, for Blacks, there were step reductions in mortality at 12 and 16 years of education, with constant slopes between the steps. For Whites, each additional year of education had a stronger effect on decreasing mortality for those with a high school diploma (65). It has been also shown that education differently predicts behaviors (68) as well as CMC among Blacks and Whites (69, 70).

The magnitude of the health gain associated with educational attainment is conditional on the availability of other resources that can be gained or purchased by the income that such education generates. Availability of social resources (19, 20, 71), as well as purchase power associated with higher SES (67, 70, 72–76), are shaped by race, place, SES, and their intersections. SES does not independently affect health (in a vacuum), and its impact is specific to the context (9, 24, 71–73, 77–81). As Marmot has mentioned, it is not just important how many resources people have, but also what they can buy with those resources (82).

In line with our findings that the protective effect of education on mortality depends on place, Stafford and Marmot found that the effect of living in a deprived area was more marked for poorer individuals. The study used individual-level data from the Whitehall II study covering health, SES, and perceived status and census data on neighborhood deprivation. Authors concluded that living in a deprived neighborhood may have the most negative health effects on poorer individuals, possibly because poor individuals are more dependent on collective resources in the neighborhood (83). Proper and colleagues also found significant interactions between neighborhood SES and level of educational attainment in the contribution of total and vigorous occupational physical activity to total physical activity. Authors concluded that neighborhood SES functions as a moderator in the relationship between individual SES and occupational physical activity (84). Another study showed that death rates among people of low SES were highest in high SES neighborhoods, lower in moderate SES neighborhoods, and lowest in low SES neighborhoods. The study showed that differences were not merely due to individual-level risk factors (28).

In the U.S., place strongly influences employment opportunities, and race impacts employer choices as well as preferences and practices of the labor market (16, 17, 85). Structural inequalities due to race and place are well documented in the U.S. labor market (86). Racial wage inequality (87) and occupational segregation based on race and place (16, 88, 89) are a well-explained phenomenon in sociology and economics literature. Different earnings of Blacks and Whites, particularly for individuals with the highest levels of education, are indicative of inequalities in how education translates to income and subsequently income to health (90). A Black man with a master’s degree in 2006 earned $27,000 less than a White man with the same education. On top of individual income, due to lower chance of marriage, being Black significantly reduces household income and wealth (91). Even with the same employment opportunity, pay for an equal job is lower for Blacks than for Whites, a phenomenon called Black–White pay gaps (92). Similar effects can be attributed to place, which shapes job opportunities through residential segregation as well as job proximity (93). In addition, Blacks attend lower quality schools, which cause a differential effect of education on employment opportunities (94). All these factors explain the differential health gain due to education and income based on race and place.

Race, place, and other SES indicators may alter the health gains associated with education (22). As explained by David Williams, the effects of race and SES are not additive but multiplicative (72). That being said, health disparities are the product of complex and non-linear interactions between race, place, and SES factors, such as education (72). Our findings that race and place interact with education but not income suggest that, although race and place alter the health gains associated with education, income is universally protective, regardless of the context.

The results should be interpreted in the light of at least four limitations. First, education and income are both subject to change over time; however, we only measured their baseline status. Although this is likely a minor issue for education, income tends to change more over time. Second, our education measure is not on educational credentials but years of education completed. These two limitations cause measurement bias and may have impacted our magnitude of the association between education, income, and mortality. Third, our sample size was not equal based on race and place. As a result, statistical power may differ across groups. Fourth, due to the different distribution of education based on race and place, we could not use multiple education cut-offs and, thus, only used high school graduation. Despite the above limitations, this study extended the literature by showing that race and place alter long-term effects of education but not income on mortality in a nationally representative cohort.

To conclude, the protective effects of education but not income on mortality in the U.S. depend on race and place (i.e., urbanity). The lower health gain of educational attainment for Blacks compared to Whites is possibly due to structural racism, which even blocks gains associated with education for Blacks. Future research should explore mechanisms by which race and place alter the effects of health return associated with education in the U.S.

## Informed Consent

All procedures followed were in accordance with the ethical standards of the responsible committee on human experimentation (institutional and national) with the Helsinki Declaration of 1975, as revised in 2000. Informed consent was obtained from all participants included in the study. The study protocol received approval by the Institutional Review Board of the University of Michigan.

## Animal Studies

No animal studies were carried out by the authors for this article.

## Author Contributions

SA designed the study, analyzed the data, and contributed to the revision. ML drafted the manuscript and revised the paper.

## Conflict of Interest Statement

The authors declare that the research was conducted in the absence of any commercial or financial relationships that could be construed as a potential conflict of interest.

## Footnotes

^1^Assari S. Racial disparities in the link between education and mortality in United States. *JAMA* (2016, under review).
